# Cutting-Edge Advances in Cystic Fibrosis: From Gene Therapy to Personalized Medicine and Holistic Management

**DOI:** 10.3390/genes16040402

**Published:** 2025-03-30

**Authors:** Giuseppe Fabio Parisi, Vito Terlizzi, Sara Manti, Maria Papale, Giulia Pecora, Santiago Presti, Monica Tosto, Salvatore Leonardi

**Affiliations:** 1 Pediatric Respiratory Unit, Department of Clinical and Experimental Medicine, San Marco Hospital, University of Catania, 95121 Catania, Italy; gf.parisi@policlinico.unict.it (G.F.P.); mariellapap@yahoo.it (M.P.); giupec87@hotmail.it (G.P.); santiagopresti@gmail.com (S.P.); monitosto@gmail.com (M.T.); leonardi@unict.it (S.L.); 2 Cystic Fibrosis Regional Reference Centre, Department of Paediatric Medicine, Meyer Children’s Hospital IRCCS, Viale Gaetano Pieraccini 24, 50139 Florence, Italy; 3 Pediatric Unit, Department of Human and Pediatric Pathology “Gaetano Barresi”, Azienda Ospedaliera Universitaria Policlinico “G. Martino”, University of Messina, Via Consolare Valeria, 1, 98124 Messina, Italy; sara.manti@unime.it

**Keywords:** cystic fibrosis, gene therapy, personalized therapy, CFTR, modulators

## Abstract

Cystic fibrosis (CF), a genetic disorder characterized by mutations in the CFTR gene, has seen significant advances in treatment through cutting-edge approaches such as gene therapy and personalized medicine. This review examines the current and emerging strategies shaping CF care, focusing on novel therapies that target the root cause of CF and optimize patient outcomes. CFTR modulators have transformed cystic fibrosis management by enhancing protein function for specific mutations, leading to improved lung function and quality of life. Concurrently, gene therapy offers transformative potential by aiming to correct CFTR mutations using tools like CRISPR/Cas9 or prime editing, though challenges remain in delivery and long-term efficacy. The integration of precision medicine, facilitated by genomic and computational technologies, allows for personalized treatment plans that account for genetic variability and disease severity. Complementing these approaches, holistic management emphasizes the importance of psychological support and nutritional optimization, acknowledging CF’s multi-system impact. Future directions include exploring anti-inflammatory agents and microbiome modulation to further mitigate disease morbidity. However, global disparities in treatment access continue to challenge equitable healthcare delivery, underscoring the need for policy reform and international cooperation. By synthesizing these developments, this review highlights the transformative potential of modern CF treatments, advocating for continued innovation and global healthcare equity, with the ultimate goal of dramatically improving life expectancy and quality of life for individuals with CF.

## 1. Introduction

Cystic fibrosis (CF) is a progressive, genetic disease that profoundly affects the respiratory and digestive systems, posing significant challenges in clinical management and patient care. Caused by mutations in the cystic fibrosis transmembrane conductance regulator (*CFTR*) gene, CF leads to the production of thick and sticky mucus, which can clog airways, trap bacteria, and cause persistent lung infections. This condition also affects the pancreas, liver, and intestines, complicating the digestion and absorption of vital nutrients [1].

The journey of CF research and treatment over the decades reveals a path marked by significant breakthroughs and persistent obstacles. Historically, treatments focused on managing symptoms and complications—such as airway clearance techniques and enzyme supplements—without altering the disease’s genetic underpinnings. However, the life expectancy of individuals with CF has improved dramatically, thanks in part to these supportive therapies and early interventions [2].

Over the years, advances in medical research have propelled a dramatic shift in the treatment landscape of CF. Breakthroughs in molecular biology have ushered in an era where therapies are increasingly aimed at addressing the root genetic causes of the disease. This shift is epitomized by the development of CFTR modulators, which are designed to improve the function of the defective protein [3]. These modulators offer hope for transforming CF from a fatal condition into a manageable chronic disease, significantly improving longevity and quality of life for many patients [4].

While personalized and gene therapies represent promising frontiers in the treatment of cystic fibrosis (CF), their widespread application and efficacy are still developing. The personalized approach, primarily through CFTR modulators, has made significant strides, especially for patients with specific mutations. However, its reach is limited by the diversity of mutations and variability in patient responses. Many patients either do not qualify for these therapies or do not respond optimally due to the specific mutations they carry [5,6,7].

Similarly, both gene editing and gene addition therapies face critical challenges, particularly regarding delivery mechanisms. Successful application of these therapies requires precise targeting of airway epithelial cells, which is complicated by physiological barriers such as thick mucus layers that obstruct effective delivery. While adeno-associated viruses (AAVs) are a common choice due to their ability to transduce non-dividing cells efficiently, they face limitations such as restricted cargo capacity and the potential for immune responses, which can neutralize the vector and disrupt gene expression [8]. Additionally, achieving long-term expression and avoiding insertional mutagenesis remain significant challenges with viral vectors.

Non-viral delivery systems, such as lipid nanoparticles and polymer-based nanoparticles, offer potential advantages including lower immunogenicity and the ability to carry larger genetic payloads. However, these systems require further refinement to improve their targeting specificity, stability, and overall delivery efficiency. The complexity of integrating these approaches within the hostile lung environment demands continued innovation and optimization. Addressing these delivery challenges is essential for unleashing the full potential of gene therapies in CF treatment. Overcoming these barriers will be crucial to enabling reliable correction or supplementation of defective CFTR genes across diverse patient populations, thus providing a more inclusive and effective therapeutic strategy [9].

Gene therapy, on the other hand, while showing great potential in preclinical studies, faces several hurdles before becoming a viable treatment option. Challenges include efficiently delivering the gene-editing machinery to the lung cells affected by CF, ensuring long-term expression and safety, and overcoming the body’s immune response to these therapies. Additionally, the high cost and technical complexity of developing these therapies present significant barriers to global accessibility and implementation [10,11].

Despite these challenges, research continues to push forward. Ongoing clinical trials and technological advancements aim to address these hurdles, paving the way for more effective and broadly applicable CF treatments. The journey toward fully realizing the potential of personalized and gene therapies for CF might be more extended, but the progress made so far instills hope for significant breakthroughs in the near future [12].

In this review, we explore these groundbreaking advancements, examining how emerging therapies and methodologies are reshaping the landscape of cystic fibrosis treatment. By analyzing recent scientific progress and highlighting future directions, we aim to provide a comprehensive overview of how gene therapy and personalized medicine are transforming the outlook for individuals afflicted by this challenging genetic disorder.

## 2. Methods

This review compiles and synthesizes recent advancements in CF treatment with a focus on personalized medicine and gene therapy. The methodology of this review involves several key components aimed at ensuring a comprehensive and up-to-date analysis of the current research landscape.

To capture the latest developments, a systematic search of scientific databases such as PubMed, Scopus, and Web of Science was conducted, covering the period from 2018 to 2024. Keywords used in the search included “cystic fibrosis”, “gene therapy”, “personalized medicine”, “CFTR modulators”, “CRISPR”, and “novel therapies”. Articles were selected based on their relevance, novelty, and contribution to the field of CF research. Inclusion criteria focused on peer-reviewed studies, clinical trials, and meta-analyses that provided insights into new therapeutic approaches or enhanced understanding of disease mechanisms.

For each selected article, data extraction was performed to gather information on study design, patient population, interventions, outcomes, and key findings. Emphasis was placed on studies that reported on clinical efficacy, safety, and mechanistic insights of personalized therapies and gene-editing techniques. The data extracted were systematically categorized into themes such as therapeutic innovations, genetic editing methodologies, and personalized treatment frameworks.

Each study included in the review was critically appraised using an adapted version of standard evaluation criteria, assessing the methodological rigor, validity, and potential biases. This appraisal aimed to ensure the reliability of conclusions drawn and to provide a balanced view of the current state of research.

The findings from the selected articles were synthesized thematically to provide a coherent narrative of recent advances in CF treatment. The synthesis focused on identifying current trends, emerging technologies, and future directions in CF research. Emphasis was placed on highlighting innovative approaches, potential clinical applications, and ongoing challenges in implementing these therapies.

## 3. Current State of CF Research

### 3.1. Advances in CFTR Modulators

The emergence of CFTR modulators has fundamentally transformed the treatment landscape of CF, shifting the focus from symptomatic management to targeted therapies that address the underlying genetic defects. These innovative drugs enhance the functionality of the CFTR protein, which is directly linked to the severity of CF symptoms. The modulators can be categorized into at least three main classes: potentiators, correctors, and amplifiers, each acting at different points in the CFTR protein’s life cycle [13,14,15].

Potentiators, with ivacaftor being the most notable example, were among the first to demonstrate significant clinical benefits for patients with specific CFTR gating mutations, such as G551D. Ivacaftor works by increasing the time the CFTR channel remains open at the cell surface, thereby enhancing chloride ion transport. Clinical trials have documented substantial improvements in lung function as measured by forced expiratory volume in one second (FEV1), reductions in pulmonary exacerbations, and overall enhancements in the quality of life for those taking this medication. The success of ivacaftor set the stage for a new era in the management of CF, highlighting the potential of targeted therapies based on genetic mutations [16,17].

Following the introduction of potentiators, researchers turned their attention to correctors, which assist in the proper folding and trafficking of the CFTR protein.

Lumacaftor is a well-known corrector that facilitates the proper folding and trafficking of the defective F508del CFTR protein to the cell surface. Ivacaftor, when used in combination, addresses the gating defect of the CFTR protein once it has reached the cell surface. Notably, ivacaftor’s role is not in protein correction but in enhancing channel activity, although some studies suggest that it may reduce the half-life of the rescued CFTR protein at the cell surface [18]

Although it has improved clinical outcomes for some patients, the effectiveness of lumacaftor has not been uniform across all individuals, with some experiencing only modest benefits [19,20]. The necessity for alternative and more effective correctors was emphasized, which led to the development of tezacaftor, another corrector that demonstrated a better side effect profile and greater efficacy in trials [21].

The introduction of elexacaftor represents another significant advancement in CF therapy, known primarily as a corrector. Elexacaftor works by binding to a distinct pocket on the CFTR protein, stabilizing its structure, which complements the actions of tezacaftor and lumacaftor. Elexacaftor is integrated into the ground-breaking triple-combination therapy, elexacaftor/tezacaftor/ivacaftor (ETI), which has been hailed as a landmark achievement in CF treatment. ETI represents a monumental shift in treating CF as it targets a wider range of mutations, particularly those involving the F508del mutation [22]. Clinical trials have shown marked improvements in lung function, with patients experiencing significant increases in FEV1, reductions in pulmonary exacerbations, and overall enhancements in their health-related quality of life. The real-world effectiveness of ETI continues to be validated by ongoing studies, translating clinical trial successes into everyday practice for a substantial percentage of the CF population [23,24].

The introduction of the combination of vanzacaftor, tezacaftor, and deutivacaftor represents a notable advancement in cystic fibrosis (CF) treatment options. This new triple therapy has proven effective in phase 3 trials, demonstrating non-inferiority to the established elexacaftor/tezacaftor/ivacaftor (ETI) regimen concerning FEV1 % predicted. As correctors, vanzacaftor and tezacaftor facilitate proper folding and trafficking of the CFTR protein to the cell surface. Deutivacaftor enhances the stability and activity of the CFTR protein once there. In comparison, the ETI therapy incorporates elexacaftor and tezacaftor as correctors, while ivacaftor functions as a potentiator to augment CFTR activity, increasing chloride ion transport across the cell membrane. Notably, the once-daily dosage of vanzacaftor–tezacaftor–deutivacaftor presents a potential advantage in reducing treatment burden, which may enhance patient adherence compared to the twice-daily regimen required by current modulators [25].

However, a subset of CF patients exhibit intolerance to these therapies, necessitating the exploration of alternative treatment strategies. The efficacy of CFTR modulators is highly dependent on the specific CFTR mutation. While potentiators, such as ivacaftor, primarily benefit patients with gating defects (Class III mutations), correctors, like tezacaftor, aim to improve the folding and trafficking of misfolded proteins (commonly Class II mutations, including the prevalent F508del). However, many mutations remain unresponsive to current therapies. For instance, Class I mutations, which prevent protein synthesis, and certain Class V mutations affecting protein trafficking, often show minimal response. The location and nature of the mutation within the CFTR gene significantly influence modulator efficacy [26].

Despite the transformative impact of CFTR modulators, challenges and limitations persist. It is estimated that approximately 10% of people with cystic fibrosis carry mutations that do not currently benefit from CFTR modulators. This includes individuals with Class I mutations, such as nonsense mutations, which result in premature stop codons and prevent the production of functional CFTR protein. Examples include G542X and other stop codon-generated mutations that lead to truncated protein products. These mutations typically demonstrate minimal to no response to existing modulators, which primarily target gating and folding defects (Classes II and III, respectively) [27,28].

Furthermore, certain Class V mutations, which affect the quantity of functional CFTR protein through splicing errors, also remain inadequately addressed by current treatments. These genetic variations, located in less common alleles, do not interact effectively with available CFTR-modulating drugs and require novel therapeutic approaches to address their unique pathophysiology [27,28].

The absence of effective modulators for these mutations underscores the necessity for continued research and development of new therapies that cater to this underserved patient cohort. Moreover, the clinical response to CFTR modulators can vary widely among individuals, including those sharing the same mutation. This variability is influenced by factors such as genetic background, environmental conditions, and concomitant health disorders, further complicating treatment efficacy. Understanding these variations through continued research is crucial for refining treatment strategies and optimizing care for all CF patients [29,30,31].

In addition to these challenges, access to CFTR modulators remains a significant barrier. Although these therapies have been life-changing for many, their high cost limits accessibility, particularly in low-resource settings. Ensuring equitable healthcare access necessitates collaboration among researchers, healthcare providers, and policymakers to develop strategies that make innovative therapies available to all who need them, regardless of socioeconomic status or geographic location [32].

### 3.2. Gene Therapy Developments

Gene therapy holds transformative potential for the treatment of CF by addressing the root genetic cause of the disease. Unlike traditional therapy approaches that focus on alleviating symptoms, gene therapy aims to correct or replace defective genes to restore normal function in affected tissues. The growing body of research in this field has highlighted various innovative strategies, delivery mechanisms, and ongoing challenges that must be navigated to make gene therapy a viable treatment option for CF [10,12].

One of the most promising advancements in the realm of gene therapy for CF is the development and application of gene editing technologies, particularly CRISPR/Cas9. This revolutionary tool allows researchers to make precise modifications to the genome, aiming to correct specific mutations within the CFTR gene. By directly targeting the mutation responsible for CF, CRISPR holds the potential to offer a permanent solution for certain patients [33,34,35]. Early preclinical studies using CRISPR have shown effective correction of CFTR mutations in cell lines and animal models, demonstrating the feasibility of this approach. However, the transition from laboratory research to clinical application remains complex, with challenges related to the efficient delivery of CRISPR components to the appropriate lung cells [36,37].

Although CRISPR/Cas9 has shown promise as a gene editing tool, its application to CF faces significant challenges. Efficient delivery to target lung cells remains problematic, alongside the inherent risk of off-target effects and the potential induction of immune responses. These factors require further investigation before widespread clinical application is feasible [38].

Delivery mechanisms are a critical aspect of gene therapy, as achieving effective and targeted delivery of the therapeutic agents is essential for success. A variety of viral vectors, including adeno-associated viruses (AAVs) and lentiviruses, have been explored as carriers for delivering corrected genes into the respiratory epithelium. AAVs are particularly attractive due to their ability to infect non-dividing cells and their comparatively low immunogenicity [39,40]. Recent clinical trials utilizing AAV-based gene therapy have shown promise in initial safety and efficacy results, but challenges remain in ensuring persistent expression of the CFTR protein and evading immune responses [9].

In addition to viral vectors, non-viral delivery systems such as lipid nanoparticles and electroporation technologies are being investigated. These methods may provide advantages regarding safety and simplicity of production, but further research is needed to establish their effectiveness for gene therapy in CF. Successful delivery strategies must be able to penetrate the thick mucus present in CF lungs, ensuring that therapeutic components reach the cells that require treatment [41].

Another approach involves the use of nanoparticles derived from plant-based systems or other biodegradable polymers that can enhance the stability and delivery efficiency of genetic payloads [42].

Moreover, mesoporous silica nanoparticles and other polymer-based systems offer innovative platforms for the controlled release of therapeutic genes, enabling targeted delivery to specific tissues. A comprehensive exploration of these and other non-viral techniques is essential to expand the therapeutic options available for gene therapy in CF, ultimately improving the delivery and effectiveness of these promising treatments [43].

While gene therapy holds immense potential for treating CF, integrating these approaches into standard treatment protocols presents several significant challenges that must be meticulously addressed. The long-term safety and efficacy of gene therapies remain primary concerns as they advance through clinical trials. Regulatory agencies will closely scrutinize these aspects to ensure both the immediate and persistent health benefits for patients. One of the critical challenges is mitigating the risk of off-target effects inherent in gene editing technologies. These unintended modifications at non-target sites in the genome could lead to detrimental mutations or disturb essential genetic functions. To address this, developing cutting-edge evaluation techniques, including high-resolution genome-wide sequencing, is crucial for proactively identifying and reducing these risks. Ongoing research is focusing on enhancing the specificity and fidelity of gene editing tools to minimize the possibility of such adverse effects [44].

Furthermore, the potential for immune responses presents a complex hurdle. Viral vectors, commonly used for delivering therapeutic genes, can induce immune reactions that neutralize their effectiveness, compromising the therapeutic benefits. As such, there’s a concerted effort in the development of novel vectors with improved immunogenicity profiles, alongside exploring promising non-viral delivery systems like lipid nanoparticles and polymer-based carriers. These innovations aim to circumvent immune defenses, ensuring sustained and effective gene delivery. Additionally, tailored immunosuppressive regimes could be strategically employed to facilitate better integration of these therapies [45].

Another crucial challenge relates to the efficient delivery of therapeutic constructs to specific target tissues, such as the airway epithelial cells where CFTR gene action is critical. The dense, sticky mucus in CF lungs, along with the difficulty in targeting non-dividing cells, makes achieving precise delivery inherently challenging. Developing advanced delivery techniques, such as aerosol-based systems or gene-editing gel encapsulation, is under research to enhance the penetration and specificity of gene delivery to the lung tissues. Addressing these multifaceted obstacles is vital to fully unlock the transformative potential of gene therapy in CF treatment. By continuing to refine these approaches, the field can move closer to integrating gene therapy as a viable therapeutic option, thereby expanding and enhancing the scope of personalized treatment solutions available to CF patients [44,45]. Clinical trials are emerging to investigate the safety and efficacy of gene therapy approaches in cystic fibrosis (CF) patients. These trials are crucial for assessing the real-world applicability of gene therapies and are designed to evaluate not only the immediate therapeutic effects but also the long-term outcomes and safety profiles of these innovative treatments [12]. As more data come from these trials, the scientific community gains valuable insights into how gene therapy can complement or potentially replace existing treatment regimens.

The pursuit of effective gene therapy for CF has evolved significantly beyond the initial excitement surrounding CRISPR/Cas9 technology. The focus now is on precision gene-editing tools that offer increased accuracy and reduced risk of adverse effects. Base editors represent a significant advance, enabling the targeted modification of individual DNA bases without inducing double-stranded breaks, thereby decreasing the likelihood of off-target effects [46].

Building upon this, prime editing represents a major leap in precision medicine, offering remarkable versatility and accuracy. Prime editing utilizes a modified CRISPR-Cas9 system that combines a nickase enzyme and a reverse transcriptase with a prime editing guide RNA (pegRNA). This integration allows it to achieve a wide range of genetic modifications, such as precise insertions, deletions, and all possible base-to-base conversions. The versatility of prime editing is particularly advantageous in CF, where the genetic landscape is vast and varied. This method not only offers the potential to correct mutations that base editing cannot address but also minimizes unintended genomic alterations due to its precision-targeting mechanism [47].

Despite its promise, prime editing is not without limitations. Challenges include optimizing the delivery systems to efficiently transport the prime editing components to target cells and ensuring high editing efficiency across diverse cell types. Moreover, understanding the interaction with cellular repair pathways is essential to ensure that the desired genetic changes are achieved consistently and effectively.

Current research focuses on overcoming these hurdles to harness the full potential of prime editing, with an aspiration to expand its applicability across a broader spectrum of genetic mutations found in CF patients. These advancements are crucial as we aim to integrate prime editing into clinical practice, alongside improvements in delivery technologies that ensure safe and effective application.

Another exciting area of research is the potential use of stem cell-based therapies in CF. Using induced pluripotent stem cells (iPSCs) or other stem cell types offers the possibility of generating corrected airway epithelial cells that could be reintroduced into the patient. This approach combines gene editing with cellular therapy, providing a pathway to regenerate damaged pulmonary tissue. Current investigations are ongoing to determine the feasibility of this strategy and its capacity to provide durable and effective treatment for CF [48,49,50].

In addition to traditional gene editing, RNA-based therapies offer a promising avenue for treating cystic fibrosis. These therapies utilize various types of RNA, such as mRNA or antisense oligonucleotides, to modulate or supplement CFTR protein function. Through delivery systems like lipid nanoparticles, RNA-based therapies can bypass some of the challenges associated with DNA-based approaches, such as genomic integration and potential off-target effects. By targeting the expression or splicing of CFTR mRNA, these therapies have the potential to correct the protein’s function at a post-transcriptional level, making them a versatile addition to the CF therapeutic landscape [51].

Table 1 summarizes the gene therapy approaches in CF.

## 4. Precision Medicine and Holistic Management

The movement toward precision medicine in CF extends beyond the implementation of CFTR modulators. As genomic technologies advance, a more nuanced understanding of individual patient variability from a genetic, epigenetic, and environmental perspective will emerge. Future research will focus on identifying biomarkers that can predict responses to various therapies, allowing for more tailored and effective treatment regimens [52].

Moreover, integrating artificial intelligence and machine learning into clinical decision-making processes will facilitate the analysis of vast amounts of patient data, helping clinicians predict individual responses to therapies and optimize management strategies. These advanced tools will support the development of personalized treatment plans that take into consideration a patient’s specific mutation, severity of disease, comorbidities, and treatment history [53,54].

As the understanding of CF evolves, a holistic approach to patient management will become increasingly important. The recognition that CF affects multiple organ systems necessitates a comprehensive treatment paradigm that goes beyond pharmacological interventions. Future directions in CF treatment should prioritize the integration of psychological support, nutritional guidance, and lifestyle counseling into routine care [55,56].

Research focusing on mental health in CF patients has highlighted the importance of addressing psychological well-being, as anxiety, depression, and social isolation can significantly impact adherence to treatment and overall quality of life. Programs providing mental health support, educational resources for patients and families, and peer support networks will be critical components of a holistic approach to CF management [57].

Nutritional interventions are equally vital, as maintaining optimal nutritional status can profoundly affect lung function and overall health in CF patients. Future studies will focus on optimizing dietary strategies, considering individualized nutritional needs, and expanding the availability of metabolic and enzyme replacement therapies [58].

In addition to CFTR modulators and gene therapy, several emerging therapeutic strategies hold promise for the future of CF treatment. This includes the exploration of anti-inflammatory agents to mitigate chronic lung inflammation, which significantly contributes to disease morbidity. Research into novel compounds that target inflammatory pathways in CF will be essential in managing the complex interplay between infection, inflammation, and immune response [59,60].

Another avenue involves the exploration of microbiome modulation to support pulmonary health. Growing evidence suggests that the lung microbiome plays an integral role in CF pathogenesis and disease progression. Future clinical research may explore the use of probiotics, prebiotics, or bacteriophage therapy to restore a balanced microbiome, potentially improving patients’ lung function and reducing infection risks [61,62].

Recent research highlights the significant impact that highly effective CFTR modulators, such as the ivacaftor and ET combination, have on the microbiome landscape within the CF lung environment. These treatments, by enhancing the CFTR protein function and improving airway hydration and mucus clearance, can alter the microbial milieu traditionally associated with CF airways. Clinical studies have documented shifts in the composition of lung microbiota following modulator therapy, with a noted reduction in the prevalence and density of key CF pathogens such as *Pseudomonas aeruginosa* and *Staphylococcus aureus*. These changes suggest an evolving pathogen landscape that may decrease the frequency and severity of pulmonary exacerbations [63].

Additionally, continuous development in delivery systems for existing and new therapies—such as nebulized formulations or aerosolized drug delivery—can improve drug bioavailability and patient adherence. Enhancements in technology for at-home administration will promote better management and monitoring of the disease [64].

As promising therapies continue to emerge, ensuring equitable access to these innovations remains a critical challenge. The disparity in healthcare resources across different regions underscores the need for strategies that facilitate global access to effective CF treatments [65].

Future initiatives should aim to reduce the costs of emerging therapies through collaboration between pharmaceutical companies, governments, and health organizations. Advocating for policy changes that prioritize affordability and accessibility of life-changing treatments will be instrumental.

Furthermore, enhancing education and training for healthcare providers in underserved regions will improve the standard of care for CF patients. Global partnerships and initiatives can facilitate resource sharing, clinical trial access, and telehealth solutions to extend specialized care to remote areas. Table 2 summarizes the future directions in precision medicine and holistic management of CF.

## 5. Conclusions

The advancements in cystic fibrosis (CF) treatment herald a new era of hope and promise for affected individuals. CFTR modulators have transformed the management of the disease by directly targeting the underlying genetic defects, significantly improving lung function and quality of life for many patients. The introduction of progressive therapies, particularly triple-combination treatments, has expanded access and efficacy for a broader patient population.

Similarly, gene therapy stands at the forefront of potential curative approaches, with innovative techniques like CRISPR/Cas9 or prime editing offering the possibility of correcting CFTR mutations at their source. Continued research will be essential to optimize delivery methods and ensure long-term safety.

The future of CF treatment will increasingly embrace precision medicine, customizing therapies based on individual genetic profiles, and enhancing holistic care that includes mental health support and nutritional management.

However, challenges remain, particularly regarding equitable access to these advances around the globe. Efforts must focus on reducing costs, improving access for underserved populations, and advocating for healthcare policies that promote equitable treatment.

In conclusion, the progress in CF research and treatment signals a promising future. Through ongoing collaboration among researchers, clinicians, and policymakers, we can transform cystic fibrosis into a manageable chronic condition, ultimately improving the quality of life for those living with the disease.

## Figures and Tables

**Table 1:** Summary of gene therapy approaches in cystic fibrosis.

Gene Therapy Approach	Mechanism	Type of Delivery	Status of Research	Potential Benefits
CRISPR Cas9	Precise editing of CFTR gene mutations	Typically uses viral vectors (e.g., AAV)	Preclinical studies showing efficacy	Permanent correction of specific mutations
Base Editing	Direct conversion of one DNA base to another	Viral vectors or non-viral systems	Emerging research; early-phase trials	High precision with reduced off-target effects
Prime Editing	Targeted insertions, deletions, and base conversions	Viral vectors or non-viral systems	Early experimental studies	Versatile editing capacity with high accuracy
RNA-Based Therapy	Use of RNA to modify or supplement CFTR protein	Lipid nanoparticles or other carriers	Developing	Multiple aspects of CFTR function can be targeted

**Table 2:** Future directions in precision medicine and holistic management of cystic fibrosis.

Component	Description	Importance	Future Research Focus
Genomic technologies	Advancements in understanding genetic, epigenetic, and environmental variability	Enables tailored treatment regimens	Identifying biomarkers to predict therapy responses
Artificial intelligence and machine learning	Integration into clinical decision-making processes	Enhances analysis of patient data for individualized care	Optimizing personalized treatment plans
Holistic patient management	Comprehensive approach that includes physical, psychological, and nutritional aspects	Recognizes CF’s impact on multiple organ systems	Incorporating psychological support and counseling
Mental health support	Programs focusing on anxiety, depression, and social isolation	Improves treatment adherence and quality of life	Developing educational resources and peer support networks
Nutritional interventions	Optimization of dietary strategies for cystic fibrosis patients	Essential for maintaining nutritional status and lung function	Expanding access to metabolic and enzyme replacement therapies
Emerging therapeutic strategies	Exploration of anti-inflammatory agents and microbiome modulation	Addresses chronic inflammation and microbial health	Research into novel compounds targeting inflammatory pathways
Microbiome modulation	Use of probiotics, prebiotics, or bacteriophage therapy to restore a balanced microbiome	Integral to cystic fibrosis pathogenesis and lung health	Investigating effects on lung function and infection reduction
Delivery systems	Development of nebulized formulations or aerosolized delivery methods	Improves drug bioavailability and patient adherence	Enhancing at-home administration technologies
Global access	Strategies to ensure equitable access to therapies worldwide	Addresses disparities in healthcare resources	Collaborating to reduce therapy costs and advocate for accessible treatments
Education for healthcare providers	Training for providers in underserved regions	Improves the standard of care for cystic fibrosis patients	Focus on clinical trial access and telehealth solutions

## Data Availability

Not applicable.
